# Predictors and outcomes of early post-operative veno-arterial extracorporeal membrane oxygenation following infant cardiac surgery

**DOI:** 10.1186/s40560-018-0326-4

**Published:** 2018-09-03

**Authors:** Gabriela A. Kuraim, Daniel Garros, Lindsay Ryerson, Fahimeh Moradi, Irina A. Dinu, Gonzalo Garcia Guerra, Diane Moddemann, Gwen Y. Bond, Charlene M. T. Robertson, Ari R. Joffe, For the Western Canadian Complex Pediatric Therapies Follow-up Program

**Affiliations:** 1 Division of Pediatric Critical Care, Department of Pediatrics, University of Alberta, 4-546 Edmonton Clinic Health Academy, 11405 87 Avenue, Edmonton, Alberta T6G 1C9 Canada; 2 School of Public Health, University of Alberta, Edmonton, Canada; 3 Department of Pediatrics and Child Health, University of Manitoba, Winnipeg, Canada; 4 Glenrose Rehabilitation Hospital, Edmonton, Canada; 5 Division of Developmental Pediatrics, Department of Pediatrics, University of Alberta, Edmonton, Canada

**Keywords:** Cardiac surgery, Extracorporeal membrane oxygenation, Outcomes research, Pediatric intensive care units, Predictor

## Abstract

**Background:**

We aimed to determine predictors of, and outcomes after, veno-arterial extracorporeal membrane oxygenation instituted within 48 h after cardiac surgery (early ECMO) in young infants.

**Methods:**

Patients ≤ 6 weeks old having cardiac surgery from 2003 to 2012 were enrolled prospectively. Patients cannulated pre-operatively, intra-operatively, or ≥ 48 h post-operatively were excluded. Variables at *p* ≤ 0.1 on univariate regression were entered into multiple logistic regression to predict early ECMO. Early-ECMO cases were matched 1:2 for six demographic variables, and death by age 2 years old (determined using conditional logistic regression; presented as odds ratio (OR), 95% confidence interval (CI)) and General Adaptive Composite scores at age 2 years (determined using Wilcoxon rank sum) were compared; *p* ≤ 0.05 was considered statistically significant.

**Results:**

Of 565 eligible patients over the 10-year period, 20 had early ECMO instituted at a mean (standard deviation) of 12.4 (11.4) h post-operatively, 10 of whom had extracorporeal cardiopulmonary resuscitation. Of early-ECMO patients, 8 (40%) were found to have residual anatomic defects requiring intervention with catheterization (*n* = 1) and/or surgery (*n* = 7). On multiple regression, the post-operative day 1 highest vasoactive-inotrope score (OR 1.02; 95%CI 1.06,1.08; *p* < 0.001), highest lactate (OR 1.2; 95%CI 1.06,1.35; *p* = 0.003), and lowest base deficit (OR 0.82; 95%CI 0.71,0.94; *p* = 0.004), CPB time (OR 1.01; 95%CI 1.00,1.02; *p* = 0.002), and single-ventricle anatomy (OR 5.35; 95%CI 1.66,17.31; *p* = 0.005) were associated with early ECMO. Outcomes at 2 years old compared between early-ECMO and matched patients were mortality 11/20 (55%) vs 11/40 (28%) (OR 3.22, 95%CI 0.98,10.63; *p* = 0.054) and General Adaptive Composite median 65 [interquartile range (IQR) 58, 81.5] in 9 survivors vs 93 [IQR 86.5, 102.5] in 29 survivors (*p* = 0.02).

**Conclusions:**

The identified risk factors for, and outcomes after, having early ECMO may aid decision making in the acute period and confirm that neurodevelopmental follow-up for these children is necessary. The hypothesis that earlier institution of ECMO may improve long-term outcomes requires further study.

## Background

Extracorporeal membrane oxygenation (ECMO) has been used to support children with congenital heart disease (CHD) since the 1970s [1, 2]. Since then, ECMO has become an accepted intervention for pediatric patients who have failed conventional medical therapy and in whom cardiac and/or respiratory insufficiency is potentially reversible [1, 2]. In addition, ECMO has been instituted as rescue during cardiopulmonary resuscitation (E-CPR) and in selected patients as a bridge to transplantation [1–3]. In the post-operative period following repair of CHD, veno-arterial ECMO may be required for (i) failure to separate from cardiopulmonary bypass (CPB), with ECMO cannulation performed in the operating room; (ii) progressive hemodynamic or hypoxic deterioration with cardiogenic shock (i.e., low cardiac output syndrome—LCOS) in the pediatric cardiac intensive care unit (PCICU); or (iii) refractory cardiac arrest occurring in the PCICU [1–3]. The Extracorporeal Life Support Organization (ELSO) has collected registry data since the 1980s and indicates that ECMO has been used for cardiac support in > 55,000 neonates and children [4]. The registry documents the survival to hospital discharge among 310 centers as follows: in neonatal cardiac ECMO 2695/6475 (42%), neonatal E-CPR 547/1336 (41%), pediatric cardiac ECMO 4265/8374 (51%), and pediatric E-CPR 1232/2996 (41%) [4]. Despite significant advances in long-term ventricular assist devices, ECMO remains the most commonly used form of mechanical cardiopulmonary support in infants and young children in the immediate post-operative period [2, 3].

In this study, we aim to (i) determine predictors of early (within 48 h) post-operative veno-arterial ECMO (early ECMO) following cardiac surgery with CPB (or without CPB, but with sternotomy for central shunt or modified Blalock-Taussig shunt (MBTS) or pulmonary artery banding (PAB)) for infants age ≤ 6 weeks and (ii) compare survival and 2-year functional outcomes of patients who had early ECMO to a matched cohort of patients who did not have early ECMO. We used 10 years of data from the interprovincial Western Canadian inception-cohort Complex Pediatric Therapies Follow-up Program (CPTFP) [5]. We hypothesized that markers of severity of post-operative illness predict early ECMO and that outcomes after early ECMO are worse than that in matched patients.

## Methods

This study is part of an interprovincial inception cohort outcomes follow-up program conducted in Western Canada, the CPTFP. Infants were identified at the time of complex cardiac surgery with CPB (or without CBP, but with sternotomy for a central shunt or MBTS or pulmonary artery banding (PAB)) and were followed prospectively [5]. Demographic, pre-operative, intra-operative, and post-operative variables that were previously agreed upon were collected prospectively. Long-term follow-up was performed, with parental or guardian consent, during follow-up visits at the tertiary site of origin. The follow-up study and database have been approved by each respective institutional health research ethics board.

In this study, we included all infants ≤ 6 weeks of age having cardiac surgery with CPB (or without CPB, but with sternotomy for central shunt or MBTS or PAB) at the Stollery Children’s Hospital from January 1, 2003, to December 31, 2012. Patients having ECMO pre-operatively, transitioned to ECMO in the operating room for failing to separate from CPB, or having ECMO instituted ≥ 48 h post-operatively were excluded. The 48-h post-operative cutoff time for defining early ECMO was chosen as we wanted to identify predictors of veno-arterial ECMO cannulation in those patients who deteriorate early after cardiac surgery and who therefore might have been considered for ECMO cannulation in the operating room (prior to admission post-operatively).

The CPTFP records data prospectively post-operatively on day 1 (defined as the day of surgery). In this study, the acute-care data on day 1 post-operatively did not include values in the 2 h immediately preceding ECMO cannulation or values after ECMO cannulation. The values within 2 h of cannulation are likely to occur when patients are in extremis and thus do not reflect those values that would lead to the decision to cannulate (i.e., are not useful to predict in advance which patients may need ECMO). Acute-care data for patients who had early ECMO were retrospectively confirmed by chart review to be those at least 2 h prior to ECMO cannulation. These variables included the following: highest lactate, highest vasoactive-inotrope score [6], highest epinephrine dose, lowest pH, lowest base deficit, lowest PaO2, highest creatinine, open sternum, steroids started for blood pressure, dialysis, and seizure. We also collected other operating room and post-operative day 1 variables for the early-ECMO patients for descriptive purposes, including pediatric risk of mortality (PRISM III) score and risk adjustment for congenital heart surgery (RACHS-1) category, intra-operative inotrope use and lactate, indication for ECMO, and investigations done peri-operatively (transesophageal echocardiogram (TEE) and cardiac catheterization).

Over the 10-year time period of this study, we have had 4 pediatric cardiac surgeons operating and 11 cardiac intensivists. Clinical practice of the surgeons and intensivists is at their discretion, and we do not have specific protocols for management. Post-operative management is up to the bedside clinicians, usually a collaboration between the cardiac intensivists, cardiac surgeons, and cardiologists, who round each morning as a group on the cardiac patients in the PCICU. The preferred initial vasoactive agent for low cardiac output is epinephrine, and steroids are considered in moderate to severe low cardiac output syndrome (LCOS). Over the years, practices may have changed with emerging research, although we did not find that year of surgery was a predictor of early ECMO (Table 1). Some of the practice, including catecholamine and steroid use, are given in the “Results” section.

**Table 1:** Description of the cohort of patients having, and not having, ECMO within 48 h of surgery from years 2003–2012

Variable	All patients *n* = 565	Early-ECMO patients *n* = 20	Non-ECMO patients *n* = 545	*p* value^a^
Demographic variables				
Sex (male)	350 (62%)	13(65%)	337(62%)	0.775
Birth gestation (weeks)	38.7(1.8)	38 (1.7)	39 (1.8)	0.420
Year of treatment	2007.5 (3.2)	2008 (3.4)	2007 (3.2)	0.185
Chromosomal abnormality	54(9.6%)	2(10.0%)	52(9.5%)	0.945
Single-ventricle anatomy	191(34%)	13(65%)	178(33%)	0.003
Antenatal diagnosis	278(49%)	12(60%)	266(49%)	0.317
Birth weight (grams)	3229.4(608.4)	3121.5(542.2)	3233(611.0)	0.420
Socioeconomic index (in survivors)	(*n* = 474) 42.5(14.4)	(*n* = 9) 47.8(21.1)	(*n* = 465) 42.4(14.2)	0.473
Mother’s years of schooling (in survivors)	(*n* = 474) 13.4(2.7)	(*n* = 9) 13.4(3.2)	(*n* = 465) 13.4(2.7)	0.996
Pre-operative variables				
Ventilator days	5.4(6.2)	7.1(5.1)	5.3(6.2)	0.198
Highest creatinine (μmol/L)	63.3(28.1)	73.8(37.7)	63(27.7)	0.099
Highest lactate (mmol/L)	3.3(3.3)	4.5(4.2)	3.3(3.2)	0.093
Highest vasoactive-inotrope score	5.8(13.5)	4.5(7.8)	5.8(13.7)	0.661
Highest epinephrine dose (μg/kg/min)	0.02(0.11)	0.02(0.04)	0.02(0.11)	0.979
Age at time of surgery (days)	13.6(10.7)	12.6(8.7)	13.7(10.8)	0.658
Operating room variables				
Cardiopulmonary bypass time (minutes)	113(49.0) *n* = 519	142.4(80.9) *n* = 19	111.8(47.4) *n* = 500	0.118
Aortic cross-clamp time (minutes)	53(26) *n* = 516	63(41) *n* = 19	53(25) *n* = 497	0.254
Deep hypothermic circulatory arrest time (minutes)	20(13.6) *n* = 377	27.3(19.0) *n* = 16	19.8(13.2) *n* = 361	0.040
Deep hypothermic circulatory arrest used	377(67%)	16(80%)	361(66%)	0.273
Need to reinstate cardiopulmonary bypass	40(8%)	4 (20%)	36(7%)	0.055
Post-operative day 1 variables				
Highest lactate (mmol/L)	5.1(2.7)	8.2(4.7)	5.0(2.6)	0.008
Highest vasoactive-inotrope score	12.3(13.6)	36.6(36.2)	11.4(11.1)	0.005
Highest epinephrine dose (μg/kg/min)	0.09(0.13)	0.21(0.26)	0.09(0.12)	0.049
Lowest pH (0.1 units)	7.30(0.07)	7.26(0.07)	7.30(0.07)	0.017
Lowest base deficit	− 2.5(3.2)	− 5.1(3.7)	− 2.3(3.2)	< 0.001
Lowest PaO2 (mmHg)	53.3(20.6)	50(35.3)	53.4(20.0)	0.729
Highest creatinine (μmol/L)	53.67(17)	63.3 (28)	53.3 (16)	0.127
Open sternum from operating room	308(55%)	17(85%)	291(53%)	0.005
Steroids started for blood pressure	108(19%)	6(30%)	102(19%)	0.243
Dialysis	9(1.6%)	2(10.0%)	7(1.3%)	0.002
Seizure at any peri-operative time	39(6.9%)	4(20.0%)	35(6.4%)	0.042
Outcomes				
Death by 30 days	12(2.1%)	7(35.0%)	5(0.09%)	< 0.001
Death by 2 years	75(13%)	11(55%)	64(12%)	< 0.001
Outcomes in survivors	*n* = 474	*n* = 9	*n* = 465^a^	
General Adaptive Composite score on the ABAS-II	89.8 (18.6)	69.7(13.0)	90.3(18.4)	0.001
General Adaptive Composite score < 70 (2 SD below mean; population expected 2.27%)	72 (15%)	5 (56%)	68 (15%)	0.005

Data are given as *n* (%), mean (standard deviation)

*ABAS* Adaptive Behavior Assessment System

^a^*t* test for continuous variables and chi-square or exact Fisher test for categorical variables

Outcomes assessments were completed at 2 years of age. Pediatric psychologists obtained the results of the Adaptive Behavior Assessment System, 2nd Edition (ABAS-II), in the patient’s respective referral institutions [7]. The preschool version of ABAS-II is a parent/caregiver-completed questionnaire to provide comprehensive, norm-referenced assessment of adaptive skills for children 0–5 years of age. The General Adaptive Composite (GAC) score from the ABAS-II is used to assess adaptive function in children, with a normative population mean of 100 and standard deviation (SD) of 15; higher scores indicate better performance, and a score < 70 is 2 SD below the mean (expected in 2.27% of the normative population).

### Statistics

Continuous variables are presented as mean (SD) and median [interquartile range, IQR] as appropriate, and categorical variables are presented as counts (percentages). The first objective was to determine predictors of early ECMO (defined as within 48 h post-operatively) following cardiac surgery at age 6 weeks or less. To screen for variables associated with this outcome, we used univariate regression models including the a priori specified variables given in Table 1. Multiple logistic regression models consisted of variables from Table 1 found approaching significance at *p* ≤ 0.10 in the univariate analysis and, after screening for multicollinearity, are presented as odds ratios (ORs) with 95% confidence intervals (CIs) and two-sided *p* values. We explored two multiple regression models: one using highest vasoactive-inotrope score and highest post-operative day 1 lactate as continuous variables, and one using these as dichotomous variables (pre-specified as highest vasoactive-inotrope score > 30 and highest lactate > 6 mmol/L). The second objective was to compare outcomes of patients who had early ECMO to a matched cohort of patients who did not have early ECMO. For each early-ECMO patient, there were two matched patients; matching was for sex, gestational age (± 2 completed weeks), year of surgery (± 2 years), chromosomal abnormality, single-ventricle physiology, and socioeconomic index (± 15 points on a scale from 17.8 to 101.7, with a mean 42.7 and SD 13) [8]. Given the six demographic variables used for matching, it was a priori decided to match 1:2 and not higher in order to avoid inability to adequately match patients. We compared the outcome of 2-year mortality using conditional logistic regression, and results are presented as OR with 95% CI and two-sided *p* values. We compared the outcome of 2-year GAC score in survivors using Wilcoxon rank sum test. Statistical analyses were performed using SAS version 9.3, and a *p* value ≤ 0.05 was considered statistically significant.

## Results

### Description of the cohort

Over the 10-year period, 585 infants less than 6 weeks of age had cardiac surgery with sternotomy. Thirteen patients were excluded as they had ECMO cannulation pre-operatively or in the operating room, and another seven excluded as they had ECMO cannulation more than 48 h post-operatively. Of the 565 patient study cohort, 545 patients did not have ECMO, and 20 patients had veno-arterial early ECMO. The demographic and peri-operative variables for all patients are given in Table 1. Among the 20 patients who had early ECMO, the mean (SD) and median [IQR] time to cannulation for ECMO was 12.4 (11.4) and 8.0 [3.1, 21.5] h post-operatively. There were 10 (50%) cannulated during E-CPR; a description of these patients and comparison between those having E-CPR and non-E-CPR are given in Table 2. The non-E-CPR ECMO patients were cannulated for LCOS (*n* = 10), sometimes associated with cardiac tamponade (*n* = 2) or hypoxia (*n* = 1); two of these patients had a post-operative cardiac arrest requiring CPR and had return of spontaneous circulation prior to ECMO cannulation. The E-CPR patients had cardiac arrest attributed to pulmonary overcirculation (*n* = 1), progressive LCOS (*n* = 5), cardiac tamponade (*n* = 1), possible pulmonary aspiration (*n* = 1), and sudden unexplained arrest (*n* = 2). The E-CPR patients had lower inotrope score on post-operative day 1, compared to the non-E-CPR ECMO patients (Table 2).

**Table 2:** Description of the patients who had early post-operative ECMO by E-CPR or non-E-CPR indication

Variable	E-CPR *n* = 10	Non-E-CPR *n* = 10	*p* value
Demographic variables			
Sex (male)	6(60%)	7(70%)	1.000
Birth gestation (weeks)	38.0 (1.9)	38.8 (1.3)	0.519
Birth weight (grams)	3141.4 (596.7)	3190.5(334.5)	0.823
Cardiac defect^a^			
RACHS-1 score	6 [4, 6]	4 [4, 6]	
HLHS	3 (30%)	3 (30%)	
HLHS variant	3 (30%)	1 (10%)	
TAPVD	1 (10%)	1 (10%)	
Pulmonary atresia	0 (0%)	1 (10%)	
Tricuspid atresia (1 with hypoplastic arch)	2 (20%)	0 (0%)	
D-TGA	1 (10%)	2 (20%)	
Other	0 (0%)	2 (20%)	
Year of admission	2009(3.6)	2008(3.2)	0.519
Age (days) at time of ECMO	13.5(10.0)	11.9(6.8)	0.679
Chromosomal abnormality	0 (0%)	2 (20%)	0.474
Operating room variables			
Cardiopulmonary bypass time (minutes)	*n* = 10	*n* = 9	0.837
	145.5(84.6)	137.6(80.6)	
Aortic cross-clamp time (minutes), *n* = 18	*n* = 10	*n* = 8	0.756
	65.0(48.8)	72.0(43.6)	
Deep hypothermic circulatory arrest time (minutes), *n* = 15	*n* = 8	*n* = 7	0.867
	24.4(19.0)	25.9(13.7)	
Highest vasoactive-inotrope score	24.9(17.4)	48.0(50.4)	0.199
Highest vasoactive-inotrope score > 30	2(20%)	4(40%)	0.628
Highest epinephrine dose (for > 30 min; μg/kg/min)	0.15(0.13)	0.37(0.50)	0.214
Highest epinephrine dose > 0.3 μg/kg/min	1(10%)	2(20%)	1.000
Highest norepinephrine dose (for > 30 min; μg/kg/min)	0.03(0.07)	0.04(0.10)	0.749
Highest dobutamine dose (μg/kg/min)	0.5(1.6)	1.0(3.2)	0.660
Highest dopamine dose (μg/kg/min)	1.9(3.0)	3.7(4.2)	0.277
Highest milrinone dose (μg/kg/min)	0.48(0.28)	1.0(2.3)	0.427
Highest lactate once off CPB	7.1(2.3)	6.3(3.5)	0.541
Lowest pH once off CPB	7.29(0.05)	7.27(0.11)	0.555
Calcium bolus given	9(90%)	8(80%)	1.000
Steroids given	9(90%)	8(80%)	1.000
Open sternum from operating room	8(80%)	9(90%)	1.000
Need to re-institute CPB	*n* = 10	*n* = 9	0.303
	3(30%)	1(11%)	
Post-operative day 1 variables			
PRISM III score	21.1 (6.5)	19.5 (6.5)	0.588
Time to ECMO after admission to PCICU (hours)	11.2(8.5)	13.7(14.2)	0.631
Highest lactate (mmol/L)	8.6(4.7)	7.7(5.1)	0.712
Highest lactate > 6 mmol/L	7(70%)	5(50%)	0.650
Lowest pH	7.28(0.05)	7.25(0.09)	0.343
Lowest base deficit	− 5.7(3.1)	− 4.4(4.1)	0.418
Lowest PaO2 (mmHg)	46.5(20.5)	54.6(47.2)	0.624
Highest creatinine (μmol/L)	61.3(25.4)	65.4(31.7)	0.753
Open sternum at any point on day 1	9(90%)	10(100%)	1.000
Highest vasoactive-inotrope score	18.4(8.8)	55.6(43.3)	0.024
Highest vasoactive-inotrope score > 30	0(0%)	7(70%)	0.003
Highest epinephrine dose (μg/kg/min)	0.10(0.06)	0.32(0.33)	0.060
Highest epinephrine dose > 0.3 μg/kg/min	0(0%)	3(30%)	0.211
Highest norepinephrine dose (μg/kg/min)	0.006(0.019)	0.06(0.10)	0.103
Highest dobutamine dose (μg/kg/min)	0	0.8(2.5)	0.343
Highest dopamine dose (μg/kg/min)	2.3(4.2)	3.2(4.9)	0.685
Highest milrinone dose (μg/kg/min)	0.50(0.36)	0.35(0.32)	0.326
Indication for ECMO			
Cardiac tamponade	–	2(20%)	
Low cardiac output syndrome	–	10(100%)	
Hypoxia	–	1(10%)	
Dysrhythmia	–	0(0%)	
Cardiopulmonary resuscitation done (excluding E-CPR)	0(0%)	2(20%)	0.474
Cardiopulmonary resuscitation time (including E-CPR; minutes)	33(6)	*n* = 2	0.797
		9(7)	
Other peri-operative period variables			
Transesophageal echocardiogram done in PCICU	6(60%)	10(100%)	0.087
Cardiac catheterization performed post-operatively	5(50%)	4(40%)	1.000
Residual defect found: requiring return to operating room or catheterization laboratory	3(30%)	5(50%)	0.650
Seizures	1 (10%)	3 (30%)	0.582
Dialysis	3(30%)	5(50%)	0.650
Steroids started for low cardiac output syndrome prior to ECMO	3(30%)	7(70%)	0.179
Outcomes			
Duration of ECMO (hours)	282(304)	207(125)	0.484
Peri-operative ventilation days	46(30)	46(76)	0.994
Intensive care days post-operatively	38(137)	33(46)	0.782
Total hospital days	73(52)	42(41)	0.154
Death < 10 days post-operatively	1(10%)	3(30%)	0.582
Death ≤ 30 days post-operatively	2(20%)	5(50%)	0.350
Death by hospital discharge	3(30%)	5(50%)	0.650
Death by 2 years of age	4 (40%)	7 (70%)	0.178
General Adaptive Composite score on the ABAS-II	*n* = 6	*n* = 3	0.756
	69.8 (14.3)	69.3 (12.5)	
General Adaptive Composite score < 70 on ABAS-II	4 (66%)	1 (33%)	0.961

Data are given as *n* (%), mean (standard deviation). *p* value is for Fisher’s exact test (categorical data) or independent samples two-sided *t* test (continuous data)

*ABAS* Adaptive Behavior Assessment System, *CPB* cardiopulmonary bypass, *E-CPR* extracorporeal cardiopulmonary resuscitation, *ECMO* extracorporeal membrane oxygenation, *HLHS* hypoplastic left heart syndrome, *LVOTO* left ventricle outflow tract obstruction, *PCICU* pediatric cardiac intensive care unit, *PRISM III Score* pediatric risk of mortality score, *RACHS-1* risk adjustment for congenital heart surgery score, *TAPVD* total anomalous pulmonary venous drainage, *TGA* transposition of the great arteries, *VSD* ventricular septal defect

^a^Surgery performed was as follows: Norwood-Sano for *n* = 6 HLHS, *n* = 2 HLHS variants, and *n* = 1 tricuspid atresia; Norwood-BT for *n* = 1 HLHS variant; TAPVD repair for *n* = 2 TAPVD; right ventricle to pulmonary artery conduit with closure of VSD for *n* = 1 pulmonary atresia; central shunt for *n* = 1 HLHS variant; pulmonary artery banding for *n* = 1 tricuspid atresia; arterial switch with repair of VSD, or atrial septal defect and LVOTO, or atrial septal defect, VSD, right ventricle muscle bundles, and LVOTO for *n* = 3 d-TGA; right ventricle outflow tract obstruction resection and pulmonary valve plasty for *n* = 1 poly-valvular disease with Noonan’s syndrome; Ross-Konno procedure for *n* = 1 with complex LVOTO (sub-aortic stenosis, aortic valve stenosis, hypoplastic aortic arch with coarctation)

The early-ECMO patients were investigated with TEE in 16 (80%) and cardiac catheterization in 9 (45%) post-operatively, with 8 (40%) found to have residual anatomic defects requiring intervention with catheterization (*n* = 1) and/or surgery (*n* = 7). Of the 8 with residual anatomic defects, 5 had an intra-operative TEE, 3 of which detected the residual lesion that later worsened post-operatively, and 2 of which missed the residual lesion. Catheter interventions included bilateral stenting of stenotic pulmonary arteries after ECMO decannulation; one other patient had dilation of a coarctation of the aorta 3 months after ECMO decannulation. Surgical interventions included clipping a central shunt due to overcirculation (followed by a right pulmonary artery band after decannulation), Sano revision due to coronary artery compression, left coronary re-implantation due to kinking, left coronary osteoplasty due to stenosis, MBTS and pulmonary valve replacement, left ventricle laceration repair, and neoaortic valve repair, all done during the ECMO period; one other patient had patch augmentation of the ascending aorta 3 months after ECMO decannulation. Three early-ECMO patients had a heart transplant prior to hospital discharge, one of whom had the transplant prior to ECMO decannulation.

### Predictors of early (within 48 h) post-operative ECMO

**Fig. 1:**
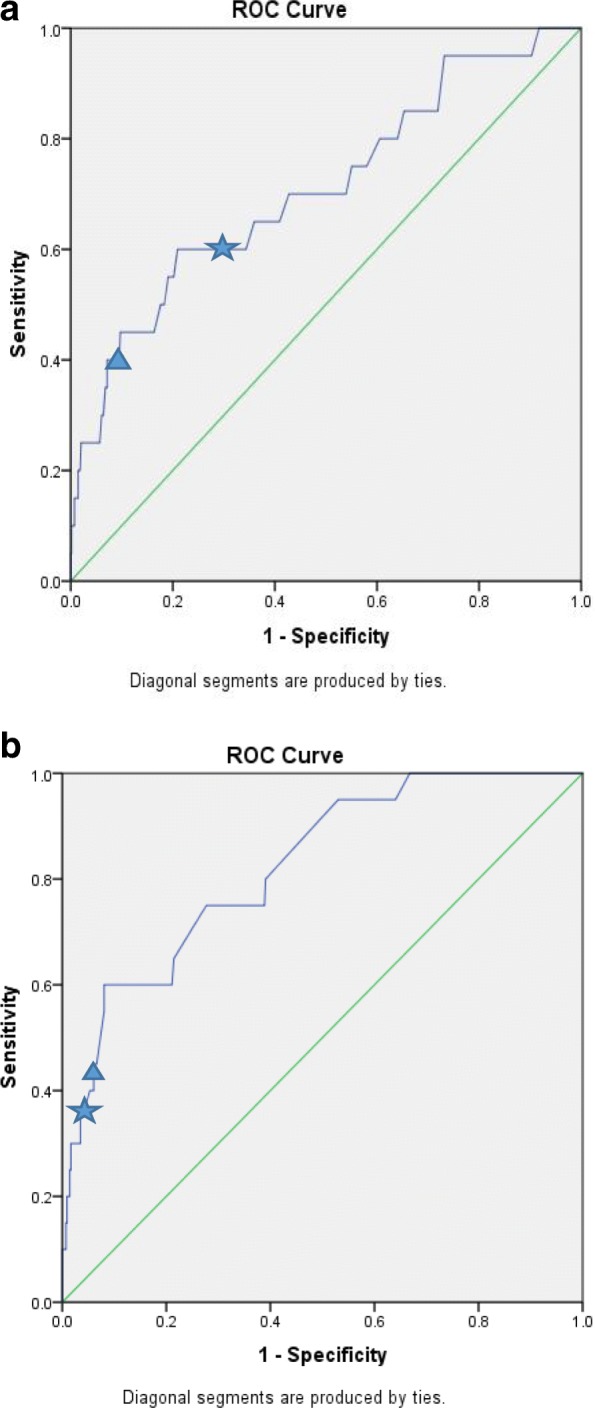
Association of day 1 post-operative highest lactate and vasoactive-inotrope score with early ECMO. Receiver operating curves for day 1 post-operative highest **a** lactate and **b** inotrope score association with early ECMO. Area under the curve is 0.709 (95% CI 0.581–0.838) and 0.823 (95% CI 0.734–0.911), respectively. The star indicates highest lactate > 6 mmol/L, highest vasoactive-inotrope score > 30; the triangle indicates highest lactate > 9 mmol/L, highest vasoactive-inotrope score > 27

Comparison of the early-ECMO (*n* = 20) to non-ECMO (*n* = 545) patients is shown in Table 1. Those variables shown in Table 1 that on univariate analysis had a *p* value ≤ 0.10 were entered into the multiple logistic regression (Table 3). In multiple regression, highest vasoactive-inotrope score (as a continuous or dichotomous variable), highest post-operative day 1 lactate (as a continuous and dichotomous variable), and lowest base deficit, single-ventricle physiology, and CPB time were statistically significantly independently associated with early ECMO. A vasoactive-inotrope score > 30 occurred in 7/20 (35%; sensitivity 35%) of those having early ECMO and 21/545 (4%; specificity 96%) of those not (*p* < 0.001); lactate > 6 mmol/L in 12/20 (60%; sensitivity 60%) of those having early ECMO and 153/545 (28%; specificity 72%) of those not (*p* = 0.002); and both thresholds were crossed in 4/20 (20%; sensitivity 20%) of those having early ECMO and 8/545 (1.5%; specificity 98.5%) of those not (*p* < 0.001). These proportions were similar in patients with single-ventricle and biventricular physiology. Single-ventricle patients with and without early ECMO had vasoactive-inotrope score > 30 in 4/13 (31%) vs. 8/178 (5%) (*p* = 0.005), lactate > 6 mmol/L in 9/13 (69%) vs. 78/178 (44%) (*p* = 0.09), and both in 2/13 (15%) vs. 3/178 (1.7%) (*p* = 0.028). Biventricular patients with and without early ECMO had respective values of 3/7 (43%) vs. 13/367 (4%) (*p* = 0.002), 4/7 (57%) vs. 75/367 (20%) (*p* = 0.039), and 2/7 (29%) vs. 4/367 (1.4%) (*p* = 0.006). Receiver operating curves for day 1 highest lactate (area under the curve (AUC) 0.71, 95% CI 0.58–0.84) and highest vasoactive-inotrope score (AUC 0.82, 95% CI 0.73–0.91) are shown in Fig. 1. In Fig. 1, we show two thresholds: our pre-specified thresholds of highest lactate > 6 mmol/L and highest vasoactive-inotrope score > 30, and a post hoc threshold of highest lactate > 9 mmol/L and highest vasoactive-inotrope score > 27.

**Table 3:** Univariate and multiple logistic regressions for having ECMO instituted in the first 48-h post-operative cardiac surgery

Variables	Univariate OR (95% CI)	*p* value	Multiple regression model 1^a^ OR (95% CI)	*p* value	Multiple regression model 2^a^ OR (95% CI)	*p* value
Highest vasoactive-inotrope score day 1	1.05 (1.03, 1.07)	< 0.001	1.04 (1.02, 1.06)	< 0.001		
Highest vasoactive-inotrope score > 30 day 1	13.44 (4.76, 33)	< 0.001			12.1 (4.25, 34.4)	< 0.001
Highest lactate day 1 (mmol/L)	1.30 (1.16, 1.46)	< 0.001	1.2 (1.06, 1.35)	0.003		
Highest lactate day 1 > 6 mmol/L	4.76 (1.85, 12.5)	0.001			3.45 (1.34, 8.91)	0.010
Lowest base deficit day 1	0.80 (0.70, 0.90)	< 0.001	0.82 (0.71, 0.94)	0.004	0.80 (0.70, 0.91)	0.001
Single-ventricle anatomy	3.83 (1.50, 9.76)	0.005	5.35 (1.66, 17.31)	0.005	3.88 (1.35, 11.15)	0.012
Open sternum from operating-room	7.85 (1.81, 34.17)	0.006				
Highest epinephrine dose day 1 (0.1 μg/kg/min)	13.44 (4.86, 37.15)	0.008				
Cardiopulmonary bypass time (minutes)	1.01 (1.00, 1.02)	0.010	1.01 (1.00, 1.02)	0.002	1.01 (1.00, 1.02)	0.020
Highest creatinine day 1 (μmol/L)	1.03 (1.01, 1.05)	0.010				
Post-operative dialysis	8.54 (1.66, 44.03)	0.010				
Lowest pH day 1 (0.1 unit)	0.00 (0.00, 0.00)	0.010				
Deep hypothermic circulatory arrest time (minutes)	1.03 (1.01, 1.06)	0.010				
Need to re-institute cardiopulmonary bypass	3.72 (1.30, 10.62)	0.014				
Aortic cross-clamp time (minutes)	1.01 (1.00, 1.03)	0.100				

Data are given as odds ratio (OR) and 95% confidence interval (95% CI)

^a^Two multiple regressions are shown: with inotrope score and lactate as continuous measures (model 1) and with inotrope score and lactate as dichotomous measures (model 2). Dialysis is not used in multiple regressions, as too few patients had dialysis

### Outcomes of early (within 48 h) post-operative ECMO and matched comparison patients

Comparison of the early-ECMO patients (*n* = 20) to the matched patients (*n* = 40) is given in Table 4. As expected, on univariate analysis, early-ECMO patients had higher post-operative day 1 highest lactate and vasoactive-inotrope score, and lower post-operative day 1 base deficit (Table 4). Mortality at 30 days and 2 years in early-ECMO and matched patients was 7/20 (35%) vs. 2/40 (5%), and 11/20 (55%) vs. 11/40 (28%), respectively. On conditional logistic regression, mortality at 2 years was higher in the early-ECMO patients, OR 3.22 (95% CI 0.98, 10.63; *p* = 0.054). In the 9 early-ECMO survivors and 29 matched patient survivors, GAC at 2 years was median 65 [IQR 58, 82] vs. 93 [IQR 87, 103]. By Wilcoxon rank sum test, GAC at 2 years was significantly lower in the early-ECMO patients (*p* = 0.02). Of early-ECMO and matched patient survivors, GAC < 70 occurred in 5/9 (56%) vs. 3/29 (10%), respectively.

**Table 4:** Description of the early-ECMO patients and the matched non-ECMO patients, 2003–2012

Variable	All patients (*n* = 60)	Early-ECMO patients (*n* = 20)	Non-ECMO matched patients (*n* = 40)	*p* value
Matching variables				
Sex (male)	39(65%)	13(65%)	26(65%)	1.000
Single-ventricle anatomy	39(65%)	13(65%)	26(65%)	1.000
Birth gestation (weeks)	38.5(1.6)	38(1.7)	38.7(1.5)	0.176
Year of treatment	2008.2(3.4)	2008.4(3.4)	2008.1(3.4)	0.747
Chromosomal abnormality	6(10%)	2(10%)	4(10%)	1.000
Socioeconomic index	*n* = 38	*n* = 9	*n* = 29	0.522
	44.0(15.0)	47.8(2.1)	42.9(12.6)	
Demographic variables				
Antenatal diagnosis	41(68.3%)	12(60%)	29(72.5%)	0.384
Birth weight (grams)	3227.3(523.8)	3121.5(542.2)	3280(513.1)	0.272
Age at time of surgery (days)	13.1(10.4)	12.6(8.7)	13.3(11.2)	0.801
Mother’s years of schooling	*n* = 38	*n* = 9	*n* = 29	0.855
	13.6(2.5)	13.4(3.2)	13.6(2.3)	
Operating room variables				
Cardiopulmonary bypass time (minutes)	*n* = 52	*n* = 19	*n* = 33	0.091
	120.3(59.6)	142.4(80.9)	107.6(39.1)	
Aortic cross-clamp time (minutes)	*n* = 52	*n* = 19	*n* = 33	0.301
	56.5(30.2)	63.3(41.3)	52.5(21.2)	
Deep hypothermic circulatory arrest time (minutes)	*n* = 47	*n* = 16	*n* = 31	0.304
	24(16)	27(19)	22(14)	
Deep hypothermic circulatory arrest used	47(81%)	16(80%)	31(81.6%)	1.000
Need to re-institute cardiopulmonary bypass	5(9%)	4(20%)	1(2.8%)	0.050
Post-operative day 1 variables				
Highest lactate (mmol/L)	6.3(4.0)	8.2(4.8)	5.4(3.3)	0.027
Highest vasoactive-inotrope score	22.1(27.3)	36.7(36.1)	14.9(18.1)	0.017
Highest epinephrine dose (μg/kg/min)	0.18(0.31)	0.21(0.26)	0.16(0.34)	0.579
Lowest pH	7.28(0.07)	7.26(0.07)	7.30(0.07)	0.100
Lowest base deficit	− 3.3(3.3)	− 5.1(3.7)	− 2.5(2.7)	0.008
Highest creatinine (μmol/L)	59.6(23.2)	63.3(28.0)	57.6(20.4)	0.373
Open sternum from operating room	44(73%)	18(90%)	26(65%)	0.062
Heart transplant at any time	5 (8%)	3 (15%)	2 (5%)	0.322
Outcomes				
Death by 2 years	22(36.7%)	11(55.0%)	11(27.5%)	0.049
Death by 30 days	9(15%)	7(35%)	2(5%)	0.004
General Adaptive Composite on the ABAS-II	*n* = 38	*n* = 9	*n* = 29	0.002
	85.6 (18.3)	69.7 (12.9)	90.5 (16.9)	
General Adaptive Composite < 70 (2 SD below mean; populations expected 2.27%)	8/38 (21%)	5/9 (56%)	3/29 (10%)	0.010
Length, *z*-score at 2 years	− 0.47 (1.1)	− 0.70 (1.7)	− 0.40 (.88)	0.617
Weight, *z*-score at 2 years	− 0.55 (1.2)	− 0.93 (1.7)	− 0.43 (.98)	0.267

Data are given as *n* (%), mean (standard deviation). *p* value is for Fisher’s exact test (categorical data) or independent samples two-sided *t* test (continuous data)

*ABAS* Adaptive Behavior Assessment System

## Discussion

Over 10 years (2003–2012), the CPTFP registry included 565 patients who had cardiac surgery with sternotomy at age ≤ 6 weeks, excluding those patients who had ECMO pre-operatively, directly from the operating room, or with cannulation > 48 h post-operatively. We aimed to determine predictors of the need for early-ECMO and outcomes at 2 years of age after early ECMO. The main findings of this study include the following: First, only 20/565 (3.5%) patients were placed on veno-arterial ECMO in this early post-operative period at a mean of 12.4 (SD 11.4) h after PCICU admission. Half of these were E-CPR, and the others had LCOS (four of which were due to cardiac tamponade, dysrhythmia, or acute hypoxia). A significant proportion (8, 40%) had residual anatomic defects identified post-operatively that required catheterization or surgical intervention. Second, independent predictors of early ECMO included single-ventricle anatomy, intra-operative CPB time, and post-operative day 1 highest vasoactive-inotrope score and lactate, and lowest base deficit. These findings may help predict the need for early ECMO before emergent deterioration. However, these variables may not be discriminating enough to predict for the individual patient, even in single-ventricle patients. For example, having a vasoactive-inotrope score > 30 occurred in 31% of single ventricle and 43% of biventricular patients having early ECMO but also in 5% and 4% of those not needing early ECMO. Third, compared to a matched cohort of patients, having early ECMO was associated with higher 2-year mortality (OR 3.22, 95% CI 0.98, 10.63; *p* = 0.054) and with statistically significantly worse functional outcome measured by GAC at 2 years of age.

The 48-h post-operative time cutoff was chosen as we wanted to predict patients that deteriorate early after cardiac surgery. We are not aware of previous studies examining specifically prediction of, or outcomes of, early ECMO as defined here. A recent review found that studies of cardiac ECMO cohorts (E-CPR or not) have reported hospital survival in the range of 45–50%, with lower 2-year post-ECMO survival [3]. Consistent predictors of survival have included renal dysfunction, neurologic complication, and highest lactate prior to and during ECMO; the timing of initiation of ECMO was not examined as a predictor in most studies [3]. The few studies that did examine timing of initiation of ECMO post-operatively looked only at cannulation in the operating room vs. in the PCICU at any time post-operatively and had conflicting findings about association with survival [9–14]. The one study that examined early versus late post-operative ECMO cannulation defined these as at < 7 days vs. ≥ 7 days post-operatively and found no difference in survival [15]. Review of the ELSO database found that neonatal cardiac ECMO mortality was associated with longer time of mechanical ventilation before ECMO (< 10 h being optimal overall and < 15 h being optimal after stage 1 palliation for hypoplastic left heart syndrome); these findings suggest that early recognition and cannulation for ECMO post-operatively may be associated with improved survival [16, 17].

Whether earlier cannulation for ECMO post-operatively or avoiding deterioration to E-CPR can result in better neurologic outcomes is not known. After cardiac ECMO, neurologic outcomes with comprehensive follow-up suggest mental delay in 38% of survivors and no clear difference with E-CPR, but this is based on very small numbers [3]. Recently, the CPTFP reported a review of 10 years of cardiac ECMO in 98 patients and found 65% survival to hospital discharge, 51% survival to 5 years, full-scale intelligence quotient of survivors 79.7 (SD 16.6) with 25% below 2 SD of the population mean, and GAC of 79.2 (SD 19.4); E-CPR and location of cannulation (operating room vs. PCICU) were not associated with survival or neurocognitive outcomes [18]. Of interest, survival improved in the more recent era, and this was associated with lower lactate and inotrope scores prior to ECMO cannulation [18]. In 51 cardiac patients having E-CPR, the CPTFP reported 49% survival to hospital discharge, 43% survival to 5 years, full-scale intelligence quotient of survivors 76.5 (SD 15.9), GAC of 74.5 (SD 20.3) with 47% having a score below 2 SD of the population mean, and 24% having both full-scale intelligence quotient and GAC below 2 SD of the population mean, similar findings to those of all cardiac ECMO cases in the 10-year review [19]. When the CPTFP examined health-related quality of life, this was lower in cardiac ECMO survivors than in other children with CHD having surgery in early infancy; whether cannulation was from the operating room or done in the PCICU was not associated with this outcome [20]. Finally, of 502 CPTFP patients having cardiac surgery early in infancy, ECMO was an independent predictor of mortality at 4.5 years (hazard ratio 1.93, 95% CI 1.17, 3.18, *p* = 0.010) and of neurocognitive outcome, with an effect size for full-scale intelligence quotient of − 13.6 (95% CI − 21.3, − 5.9, *p* = 0.001) and for GAC of − 12.7 (95% CI − 20.3, − 5.0, *p* = 0.001) [21]. Although ECMO is associated with adverse outcomes, the timing of ECMO cannulation was not explored [21]. The present results are compatible with these findings from the CPTFP (including no statistically significant difference in outcomes between E-CPR and non-E-CPR early-ECMO patients) and others, and extend these findings by examining predictors and outcomes specifically of patients having early ECMO initiated in the PCICU within 48 h of surgery.

There are some important implications of the findings from this study. First, the finding that 40% of early-ECMO patients had residual anatomic lesions that were corrected by intervention using catheterization or surgery highlights the importance of patient assessment with early TEE and/or cardiac catheterization if patients are not progressing as expected. The safety and importance of TEE and cardiac catheterization to identify and manage residual lesions, particularly in patients on ECMO, has been noted by others [22–28]. Kato et al. found that patients who received catheterization within 48 h after ECMO cannulation had fewer respiratory complications and better 30-day survival than those who had later catheterization [27]. Agarwal et al. found that earlier detection of residual lesions during the first 3 days of ECMO (compared with later detection) was associated with a higher rate of decannulation and survival to hospital discharge [23]. Howard et al. found that the time to diagnosis or correction of residual lesions was significantly shorter in neonatal cardiac ECMO survivors (1 vs 2 days, *p* = 0.02) [28]. Second, the variables we found as independently predictive of early ECMO may not be easily modifiable. Vasoactive-inotrope score and base deficit reflect severity of progressive LCOS, single-ventricle anatomy is not modifiable, and CPB time is difficult to change. However, these variables may be used to help predict patients who are at high risk to need post-operative ECMO, should be on an ECMO alert, and should have an early TEE and consideration of cardiac catheterization. Avoiding E-CPR would likely also be desirable for this population. Of interest, a recent study found similar predictors of need for ECMO at any time after a Norwood operation: longer CPB time, peak lactate of 9 mmol/L within 48 h of surgery (positive predictive value 41.9%, AUC 0.83), and peak inotrope score of 27 within 48 h of surgery (positive predictive value 34.7%, AUC 0.83), remarkably similar to our findings [29]. If we use the cutoffs from this study, highest lactate of > 9 mmol/L had a sensitivity of 40% and specificity of 93%, and highest vasoactive-inotrope score of > 27 a sensitivity of 45% and specificity of 94% for early ECMO. Third, those patients having early ECMO are a high-risk group for mortality and poor functional outcomes. We have previously described that ECMO is a risk factor for adverse neurocognitive and health-related quality of life outcomes [20, 21]. These children warrant neurodevelopmental follow-up in order to identify adverse outcomes and provide early intervention in order to optimize each patient’s achievement of his/her full potential. This information may also help in counseling family when making decisions about early ECMO.

Limitations of this study should be recognized. The small number of patients having early ECMO at a single center limits the generalizability of the findings and the power of the study to determine predictors. The retrospective data collection for some variables is also a limitation; however, this was done only for objective variables recorded in the chart on the first post-operative day in early-ECMO patients. This is an observational study and therefore cannot prove causation and cannot rule out residual confounding. Finally, we must acknowledge adverse outcomes from ECMO. We hypothesize that earlier prediction of the need for, and therefore cannulation for, ECMO is desirable; however, this must be balanced against the adverse effects of unnecessary ECMO resulting from inaccurate prediction. For this reason, better prediction models may be required before our findings can direct decision making. Strengths of this study include that the majority of the data is prospectively collected by the CPTFP, with 2-year follow-up on all early-ECMO patients and 97% of non-ECMO survivors. In addition, we included a large cohort (*n* = 565) of patients having cardiac surgery in early infancy over a period of 10 years at a referral Western Canadian cardiac surgical center.

## Conclusions

Risk factors for early ECMO after cardiac surgery in young infants were identified, including single-ventricle anatomy and early markers of severity of illness (lactate, vasoactive-inotrope score, and base deficit). Early ECMO often (in 40%) indicated a residual anatomic defect requiring intervention and was associated with adverse 2-year outcomes (mortality and GAC score). Further work is necessary to improve prediction of early ECMO in larger cohorts of patients. The hypothesis that earlier institution of ECMO may improve long-term outcomes requires further study.

## Abbreviations

ABAS: Adaptive Behavior Assessment System-II
CHD: Congenital heart disease
CI: Confidence interval
CPB: Cardiopulmonary bypass
CPTFP: Complex Pediatric Therapies Follow-up Program
ECMO: Extracorporeal membrane oxygenation
E-CPR: Extracorporeal cardiopulmonary resuscitation
GAC: General Adaptive Composite
IQR: Interquartile range
LCOS: Low cardiac output syndrome
MBTS: Modified Blalock-Taussig shunt
OR: Odds ratio
PAB: Pulmonary artery banding
PCICU: Pediatric cardiac intensive care unit
SD: Standard deviation

## References

[1] Lequier L (2004). Extracorporeal life support in pediatric and neonatal critical care: a review. J Intensive Care Med.

[2] Burke CR, McMullan DM (2016). Extracorporeal life support for pediatric heart failure. Front Pediatr.

[3] Joffe AR, Lequier L, Robertson CMT (2012). Pediatric outcomes after extracorporeal membrane oxygenation for cardiac disease and for cardiac arrest: a review. ASAOI J.

[4] Thiagarajan RV, Barbaro RP, Rycus PT, McMullan M, Conrad SA, Fortenberry JD (2017). Extracorporeal life support organization registry international report 2016. ASAIO J.

[5] Robertson CM, Sauve RS, Joffe AR, Alton GY, Moddemann DM, Blakley PM (2011). The registry and follow-up of complex pediatric therapies program of Western Canada: a mechanism for service, audit, and research after life-saving therapies for young children. Cardiol Res Pract.

[6] Gaies MG, Gurney JG, Yen AH, Napoli ML, Gajarski RJ, Ohye RG (2010). Vasoactive-inotropic score as a predictor of morbidity and mortality in infants after cardiopulmonary bypass. Pediatr Crit Care Med.

[7] Harrison PL, Oakland T (2003). Manual for the adaptive behavior assessment system.

[8] Blishen BR, Carroll WK, Moore C (1987). The 1981 Socioeconomic Index for Occupations in Canada. Can Rev Soc Anthrop.

[9] Chaturvedi RR, Macrae D, Brown KL, Schindler M, Smith EC, Davis KB (2004). Cardiac ECMO for biventricular hearts after paediatric open heart surgery. Heart.

[10] Delmo Walter EM, Stiller B, Hetzer R, Alexi-Meskishvili V, Hubler M, Bottcher W (2007). Extracorporeal membrane oxygenation for perioperative cardiac support in children I: experience at the Deutsches Herzzentrum Berlin (1987–2005). ASAIO J.

[11] Alsoufi B, Al-Radi OO, Gruenwald C, Lean L, Williams WG, McCrindle BW (2009). Extra-corporeal life support following cardiac surgery in children: analysis of risk factors and survival in a single institution. Eur J Cardiothorac Surg.

[12] Pizarro C, Davis DA, Healy RM, Kerins PJ, Norwood WI (2001). Is there a role for extracorporeal life support after stage I Norwood?. Eur J Cardiothorac Surg.

[13] Ugaki S, Kasahara S, Kotani Y, Nakakura M, Douguchi T, Itoh H (2010). Extracorporeal membrane oxygenation following Norwood stage 1 procedures at a single institution. Artif Organs.

[14] Aharon AS, Drinkwater DC, Churchwell KB, Quisling SV, Reddy VS, Taylor M (2001). Extracorporeal membrane oxygenation in children after repair of congenital cardiac lesions. Ann Thorac Surg.

[15] Gupta P, DasGupta R, Best D, Chu CB, Elsalloukh H, Gossett JM (2015). Delayed extracorporeal membrane oxygenation in children after cardiac surgery: two-institution experience. Cardiol Young.

[16] Ford MA, Gauvreau K, McMullan DM, Almodovar MC, Cooper DS, Rycus PT (2016). Factors associated with mortality in neonates requiring extracorporeal membrane oxygenation for cardiac indications: analysis of the extracorporeal life support organization registry data. Pediatr Crit Care Med.

[17] Sherwin ED, Gauvreau K, Scheurer MA, Rycus PT, Salvin JW, Almodovar MC (2012). Extracorporeal membrane oxygenation after stage 1 palliation for hypoplastic left heart syndrome. J Thorac Cardiovasc Surg.

[18] Ryerson LM, Guerra GG, Joffe AR, Robertson CM, Alton GY, Dinu IA (2015). Outcomes after cardiac extracorporeal life support in children less than five years of age: a ten year cohort. Circ Heart Fail.

[19] Garcia Guerra G, Zorzela L, Robertson CMT, Alton GY, Joffe AR, Moez EK (2015). Survival and neurocognitive outcomes in pediatric extracorporeal-cardiopulmonary resuscitation. Resuscitation.

[20] Garcia Guerra G, Robertson CMT, Alton GY, Joffe AR, Moez EK, Dinu IA (2014). Health-related quality of life in pediatric cardiac extracorporeal life support survivors. Pediatr Crit Care Med.

[21] Sidhu N, Joffe AR, Doughty P, Vatanpour S, Dinu I, Alton GY (2015). Sepsis after cardiac surgery early in infancy and adverse 4.5-year neurocognitive outcomes. J Am Heart Assoc.

[22] Garg R, Murthy K, Rao S, Muralidhar K (2009). Intra-operative trans-esophageal echocardiography in congenital heart disease. Ann Card Anaesth.

[23] Agarwal HS, Hardison DC, Saville BR, Donahue BS, Lamb FS, Bichell DP (2014). Residual lesions in postoperative pediatric cardiac surgery patients receiving extracorporeal membrane oxygenation support. J Thoracic Cardiovasc Surg.

[24] Abraham BP, Gilliam E, Kim DW, Wolf MJ, Vincent RN, Petit CJ (2016). Early catheterization after initiation of extracorporeal membrane oxygenation support in children is associated with improved survival. Catheter Cardiovasc Interv.

[25] Boscamp NS, Turner ME, Crystal M, Anderson B, Vincent JA, Torres AJ (2017). Cardiac catheterization in pediatric patients supported by extracorporeal membrane oxygenation: a 15 year experience. Pediatr Cardiol.

[26] Callahan R, Trucco SM, Wearden PD, Beerman LB, Arora G, Kreutzer J (2015). Outcomes of pediatric patients undergoing cardiac catheterization while on extracorporeal membrane oxygenation. Pediatr Cardiol.

[27] Kato A, Rito ML, Lee KJ, Haller C, Guerguerian AM, Sivarajan VB (2017). Impacts of early cardiac catheterization for children with congenital heart disease supported by extracorporeal membrane oxygenation. Catheter Cardiovasc Interv.

[28] Howard TS, Kalish BT, Wigmore D, Nathan M, Kulik TJ, Kaza AK (2016). Association of extracorporeal membrane oxygenation support adequacy and residual lesions with outcomes in neonates supported after cardiac surgery. Pediatr Crit Care Med.

[29] Friedland-Little JM, Hirsch-Romano JC, Yu S, Donohue JE, Canada CE, Soraya P (2014). Risk factors for requiring extracorporeal membrane oxygenation support after a Norwood operation. J Thorac Cardiovasc Surg.

